# Sustainable Process for the Depolymerization/Oxidation of Softwood and Hardwood Kraft Lignins Using Hydrogen Peroxide under Ambient Conditions

**DOI:** 10.3390/molecules25102329

**Published:** 2020-05-16

**Authors:** Zaid Ahmad, Waleed Wafa Al Dajani, Michael Paleologou, Chunbao (Charles) Xu

**Affiliations:** 1 Institute for Chemicals and Fuels from Alternative Resources (ICFAR), Western University, London, ON N6A 5B9, Canada; zaidkahmad@gmail.com; 2 FPInnovations, 570 boul. Saint-Jean, Pointe-Claire, QC H9R 3J9, Canada; Waleed.WafaAlDajani@fpinnovations.ca

**Keywords:** kraft lignin, depolymerized kraft lignin, hydrogen peroxide, ambient temperature, hardwood, softwood

## Abstract

The present study demonstrated a sustainable and cost-effective approach to depolymerize/oxidize softwood (SW) and hardwood (HW) kraft lignins using concentrated hydrogen peroxide at temperatures ranging from 25 to 35 °C, in the absence of catalysts or organic solvents. The degree of lignin depolymerization could be simply controlled by reaction time, and no further separation process was needed at the completion of the treatment. The obtained depolymerized lignin products were comprehensively characterized by GPC–UV, FTIR, ^31^P-NMR, TGA, Py-GC/MS and elemental analysis. The weight-average molecular weights (M_w_) of the depolymerized lignins obtained from SW or HW lignin at a lignin/H_2_O_2_ mass ratio of 1:1 after treatment for 120 h at room temperature (≈25 °C) were approximately 1420 Da. The contents of carboxylic acid groups in the obtained depolymerized lignins were found to significantly increase compared with those of the untreated raw lignins. Moreover, the depolymerized lignin products had lower thermal decomposition temperatures than those of the raw lignins, as expected, owing to the greatly reduced M_w_. These findings represent a novel solution to lignin depolymerization for the production of chemicals that can be utilized as a bio-substitute for petroleum-based polyols in polyurethane production.

## 1. Introduction

The forestry and agricultural sectors worldwide are generating considerable amounts of lignocellulosic materials in the form of residues, an attractive carbon-neutral source for fuels and chemicals, which can be an economically important alternative to fossil fuels. Recently, significant advances have been made in the development of economically viable biorefineries involving the fractionation of lignocellulose into its three major constituents (i.e., cellulose, hemicellulose and lignin), and full valorization of these constituents [1]. It should be noted here that biomass is the only renewable source of carbon for chemicals and materials [2]. Kraft pulping is the dominant chemical pulping process in the world. In this process, lignin and hemicellulose in wood chips are dissolved by sodium hydroxide (NaOH) and sodium sulfide (Na_2_S) to form black liquor, which is subsequently concentrated and burned in the recovery boiler to recover chemicals and heat, both of which are crucial to the functioning of a kraft mill. In North America, however, there are many mills in which the recovery boiler is the production bottleneck with respect to pulp production. Hence, removing a portion of the lignin from the black liquor can be an effective way to offload the recovery boiler with respect to calorific load, thereby allowing for an increase in pulp production without enormous capital spending. Moreover, the isolated portion of lignin can provide an additional value-added revenue stream for the mill by valorizing lignin for bio-products.

Lignin is an aromatic heterogeneous biopolymer, with a three-dimensional, crossed-linked network structure [3]. The structural diversity of lignin arises mainly from the combination of three linked phenylpropane derivatives that are the main building blocks of lignin’s complex architecture. The three building blocks are the lignols p-coumaryl alcohol, coniferyl alcohol and sinapyl alcohol, all of which are phenylpropane (C9) units differing from each other in the substitutions at the 3- and 5-positions. These lignols are linked into a lignin aromatic network in the form of phenyl propanoids, namely p-hydroxyphenyl (H), guaiacyl (G) and syringyl (S) units, respectively. Different sources of lignin (e.g., softwood, hardwood, grasses, etc.) contain different amounts of methoxyl groups depending on how much of each of the three lignols is incorporated into the lignin macromolecules [4,5]. Technical lignins, e.g., kraft lignin extracted from the pulping process, usually have a large molecular weight and a three-dimensional, crossed-linked network structure, as described above, which makes them less reactive in applications such as, for example, chemical replacement of petroleum-based polyols in materials such as polyurethanes, due to steric hindrance [6].

It has previously been demonstrated that lignin degradation and/or de-polymerization approaches can be effective in enhancing lignin reactivity in bio-based material applications [7]. The major lignin degradation methods can be categorized according to the mechanism of lignin network depolymerization, namely, oxidative, solvolytic, hydrogenolytic or hydrolytic reactions, which are briefly reviewed below. Lignin structures were initially investigated using oxidation reactions. The types of linkages between the precursors were investigated more than 50 years ago [8]. Freudenberg et al. [9] confirmed lignin’s aromatic nature by oxidizing it with nitrobenzene in an alkaline medium at 160–180 °C for 2–3 h. In their experiments, benzaldehydes were recovered as a main product with minor amounts of benzoic acids. In this reaction, nitrobenzene was considered to act as a two-electron-accepting oxidant producing quinone methide intermediates from the phenolic units of lignin [9]. Several solvolytic depolymerization studies have been reported in the literature involving different solvent mixtures (e.g., glycerol, THF, methyl-THF and γ-valerolactone (GVL)) and different types of catalysts (e.g., oxalic acid, HCl, Lewis acids, metal chlorides and ammonia) [10,11]. Acid-catalyzed solvolytic treatment is one of the earliest techniques used to deconstruct wood components and separate lignin. Hewson et al. [12] conducted a series of treatments on maple wood meal using different combinations of acids and solvents, including HCl/ethanol and formic acid/ethylene glycol to separate lignin into water-soluble and water-insoluble components at a low temperature range. It was concluded that this approach was not sufficient to de-polymerize the complex lignin structure into monomeric/oligomeric compounds. Gasson et al. [13] investigated formic acid-catalyzed lignin depolymerization at higher temperatures in alcohol solvents. Different ratios of formic acid to ethanol were employed in a pressurized autoclave reactor. They found that methoxyphenol, catechol and phenol were obtained as the major components when the reaction temperature was raised to the 360 to 400 °C range. The primary reaction pathway for lignin degradation to form phenolic products was believed to take place via the cleavage of the β-*O*-4 bonds as catalyzed by acid [14]. Recently, Kristianto et al. [15] reported highly effective lignin depolymerization in ethanol solvent in the presence of formic acid and Ru/C. A higher formic acid content and prolonged reaction times at 350 °C in the presence of Ru/C increased the bio-oil (de-polymerized lignin) yield and reduced the oxygen content in the bio-oil owing to hydrotreatment effects of the hydrogen generated in-situ from formic acid decomposition.

In other work by the authors’ group, Mahmood et al. [16] achieved the depolymerization of kraft lignin via hydrolysis using aqueous NaOH as a catalyst. The process itself was very effective and achieved a high yield (70–90%) of depolymerized kraft lignin (DKL) with a weight-average molecular weight (M_w_) of ~1500 g/mol at 250–350 °C for 2 h. However, it was a high-pressure process with the reactor pressure ranging from 5 MPa to 16 MPa. In contrast to the above-described research work, oxidative depolymerization of lignin is a low-temperature and pressure process, offering a simpler and more energy/cost-effective approach to depolymerization of lignin at low temperatures with high product yields. Various sophisticated strategies have been reported for oxidative depolymerization of lignin model compounds or lignin, including electrochemical and photocatalytic approaches as well as approaches employing heterogeneous catalysts or ionic liquids. Valuable monomers with high selectivity were produced from lignin oxidative degradation approaches. In fact, oxidative technologies have been used for a long time for bleaching purposes in the pulp and paper industry. Some of these oxidative technologies (e.g., using chlorine) are no longer in use due to more stringent environmental regulations relating to the production of chlorinated dioxins and furans [10].

Oxidative approaches to lignin depolymerization, especially utilizing molecular oxygen, hydrogen peroxide, peroxyacids or ozone, have a great potential to become important and economically viable lignin delignification technologies [17], as the reaction pathways can yield valuable chemicals such as simple aldehydes (vanillin, syringaldehyde, p-hydroxybenzaldehyde) or acids (vanillic acid and syringic acid) [18]. Depending on the extent of oxidation, various carboxylic acids having aromatic and non-aromatic structures with mono- or di-carboxyl functional groups can be produced [19]. Hydrogen peroxide, molecular oxygen and other oxidants in the presence or absence of a catalyst have been investigated extensively. Crestini et al. reported the degradation of kraft lignin and model compounds using H_2_O_2_ as an oxidant and methylrhenium trioxide (CH_3_ReO_3_) as a catalyst, producing degraded lignin compounds containing aliphatic-OH, syringyl-OH, guaiacyl-OH, *p*-hydroxy phenyl-OH and COOH groups at room temperature [20].

Oxidative lignin depolymerization is advantageous and environmentally friendly owing to its mild reaction conditions, but it is confronted with the challenge of maintaining sufficient selectivity while avoiding over-oxidation of the substrate to gaseous products (CO or CO_2_). Another advantage of lignin oxidation is that it can result in not only depolymerization but also functionalization of complex lignin, resulting in fine chemicals bearing alcohol, aldehyde or carboxylic/dicarboxylic acid functional groups. Consequently, this exclusive feature of oxidatively depolymerized lignin (low molecular weight and functionalized) could be utilized directly for industrial applications without further modification.

Sun and Argyropoulos [21] investigated the reactivity and the efficiency of several oxidants, namely chlorine dioxide, ozone, dimethyldioxirane and alkaline hydrogen peroxide with lignin using quantitative ^31^P-NMR. They found that guaiacyl phenolic units were the most vulnerable sites in all the oxidative treatments, and chlorine dioxide and ozone were the most efficient reagents towards the formation of carboxylic acids. The elimination of condensed phenolic structures at a given reagent charge was in the order of ozone > chlorine dioxide > alkaline hydrogen peroxide. However, while ozone and chlorine dioxide must be generated on-site, hydrogen peroxide is widely available from various suppliers and is significantly less expensive than the other two oxidants. Furthermore, hydrogen peroxide is a non-toxic chemical, which decomposes to molecular oxygen and water, if not properly stabilized [22,23].

Hydrogen peroxide (H_2_O_2_) is the simplest peroxide with an oxygen–oxygen single bond. It has been widely used as an oxidizer, bleaching agent and antiseptic reagent. Concentrated hydrogen peroxide is a potent oxidizing agent due to its unstable peroxide bond. H_2_O_2_ has been commercially used for many years as an efficient bleaching reagent in the pulp and paper industry. It can effectively remove chromophoric structures present in lignin, but it is incapable of degrading the lignin structure network. To degrade lignin, H_2_O_2_ must be used together with organic acids or mineral acids [22].

Moreover, the solutions of hydrogen peroxide in water are non-ideal, as the volume of a solution is less than the sum of the volumes of the components, and there is an appreciable heat effect upon mixing. Furthermore, the vapor pressures of the solutions do not follow Raoult’s law [24].

H_2_O_2_ is a very weak acid that remains undissociated at pH < 9.0. At higher pH levels, perhydroxyl anions (HO_2_^−^) appear, and they are commonly considered to be the reactive species in oxidation reactions under alkaline conditions [25]. As is well known, the chemical composition of softwoods (SW) is different from hardwoods (HW) with respect to cellulose, hemicellulose and lignin content [26]. The type of linkages predominant in these two types of wood are shown in Table 1.

In addition, hardwoods contain mainly a mixture of syringyl (S) and guaiacyl (G) units, whereas softwoods contain mainly guaiacyl (G) units. SW and HW lignins also differ in the relative amounts of linkages between the phenylpropane units (Table 1) [29]. In the pulping delignification process, the G-based lignin structural network is more resistant to cleavage, thereby leading to a lower degree of lignin depolymerization during pulping. Essentially, the efficiency of pulping is directly proportional to the amount of syringyl (S) units in lignin [30].

Furthermore, guaiacyl (G) units have a free C-5 position, which makes them amenable to lignin re-polymerization or condensation reactions through the formation of carbon–carbon bonds [31]. As seen in Table 1, the linkage β-*O*-4 (arylglycerol-β-aryl ether) is clearly the most frequent linkage type, accounting for more than 50% of the linkages in lignin [28]. The dominant weak β-*O*-4 linkages in lignin provide opportunities for cleavage by many processes [32]. Other linkages are more resistant to chemical degradation [33].

In softwoods, the G-type lignins contain more resistant linkages with those involving the C5 of aromatic nuclei (e.g., β-5, 5–5′ and 4-*O*-5′ linkages) than S type lignins (hardwood lignins). This is the main reason for the higher frequency of C–C linkages between aromatic rings in softwood lignins than in hardwood lignins [28].

In contrast to what is reported by Kadla et al. [22], we showed that reaction with concentrated H_2_O_2_ can have a significant effect on lignin degradation in the absence of metal catalysts. Furthermore, the separation of the low molecular weight lignin product from the reaction mixture does not require the use of expensive purification processes. Hence, the process described here could set the stage for increased use of lignin in the depolymerized/oxidized form as required in various high-value applications.

## 2. Results

### 2.1. General Observations and Product Yields

**Figure 1:**
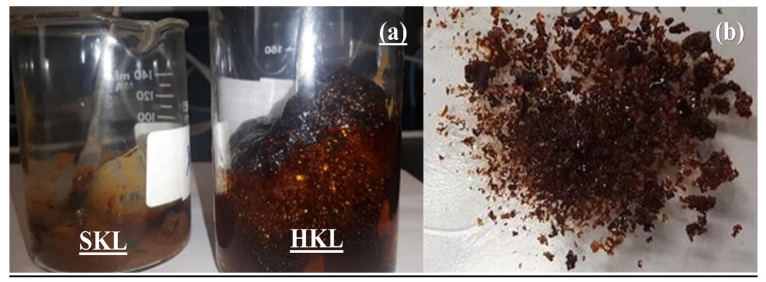
(**a**) SKL and HKL reaction mixtures during oxidative treatment with concentrated hydrogen peroxide and (**b**) depolymerized lignin sample after the treatment.

In the present work, lignin was reacted with concentrated H_2_O_2_ (50%) at ambient temperature (25 °C). The reaction needed 120 h at room temperature at 25 °C to reach completion. Hence, we accelerated the reaction by increasing the temperature to 35 °C, which reduced the reaction time to about 80 h. Reaction temperatures above 35 °C should be avoided to prevent burning the reaction slurry. A homogenous, shiny reddish powder of de-polymerized lignin product at a very high yield (>95% ± 3%) was obtained in all reaction runs (see Figure 1).

Furthermore, it was observed that during reaction of the hardwood lignin, the reaction mixture expanded and foamed, while the foaming was not significant during the softwood lignin treatment. The foam formation was likely due to the release of gases from the reaction system. The gas formation could be due to cleavage of the methoxy groups of the syringyl structures in hardwood lignin, producing methane (CH_4_). In addition, decomposition of unreacted or residual H_2_O_2_, forming O_2_ and water vapor, could also contribute to foam formation, in particular, in the presence of metal ion catalysts that might be present as impurities in lignin [34]. Homogenous powder products of de-polymerized lignins were obtained and the products had a high solubility in water and common organic solvents (e.g., acetone, ethanol, methanol, etc.).

**Figure 2:**
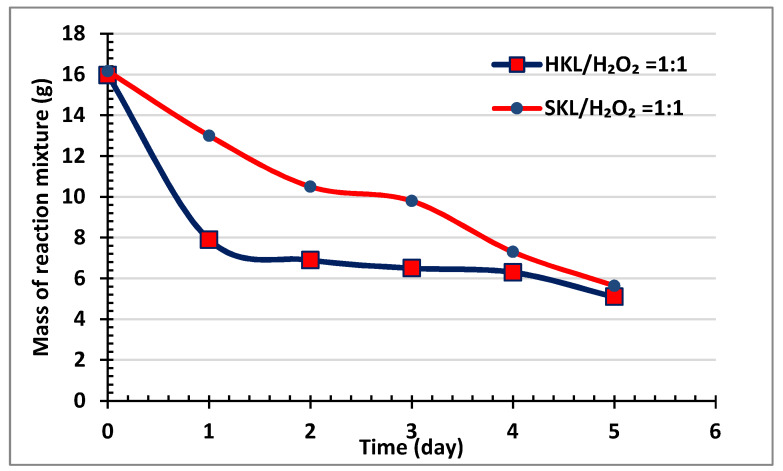
Mass of reaction mixture vs. treatment time. Note: in these tests, 5 g (d.b.) SKL or HKL and 10 g of 50 wt% H_2_O_2_ were used.

The mass loss of the reaction mixture (lignin and H_2_O_2_) vs. treatment time was monitored during the treatment where 5 g on a dry basis (d.b.) of SKL or HKL and 10 g of 50 wt% H_2_O_2_ were used, as illustrated in Figure 2. As shown in this figure, the mass loss in the case of the HKL treatment was faster than that of the SKL treatment. After 5 days, however, almost the same degree of mass loss was achieved in both treatments with HKL or SKL. The faster mass loss with HKL might be due to the higher reactivity of HKL (containing more syringyl nuclei) compared to SKL (containing more guaiacyl nuclei) towards H_2_O_2_ [35].

### 2.2. Elemental Analysis

Elemental analyses of the SKL, HKL and the depolymerized lignin products were conducted to investigate the changes in the composition of the lignins during the treatment. Table 2 presents the elemental contents (wt% on a dry basis) of C, H, N, S, ash and O of the original SKL, HKL and depolymerized lignin samples produced at various lignin/H_2_O_2_ mass ratios (1:1, 1:0.75 and 1:0.5). As seen here, the changes in H, N and S were negligible in all de-polymerization treatments with H_2_O_2_ at all mass ratios, while the carbon contents in both SKL and HKL were significantly reduced, accompanied by a marked increase in oxygen content in the depolymerized lignin products. This result suggests that the oxidative depolymerization of lignin is likely to lead to more oxygen-rich functional groups, e.g., COOH and –OH in the depolymerized lignin products, as expected. Additionally, the gases released may account for the reduction in carbon content (e.g., CO_2_, CH_4_).

### 2.3. FTIR Analysis

**Figure 3:**
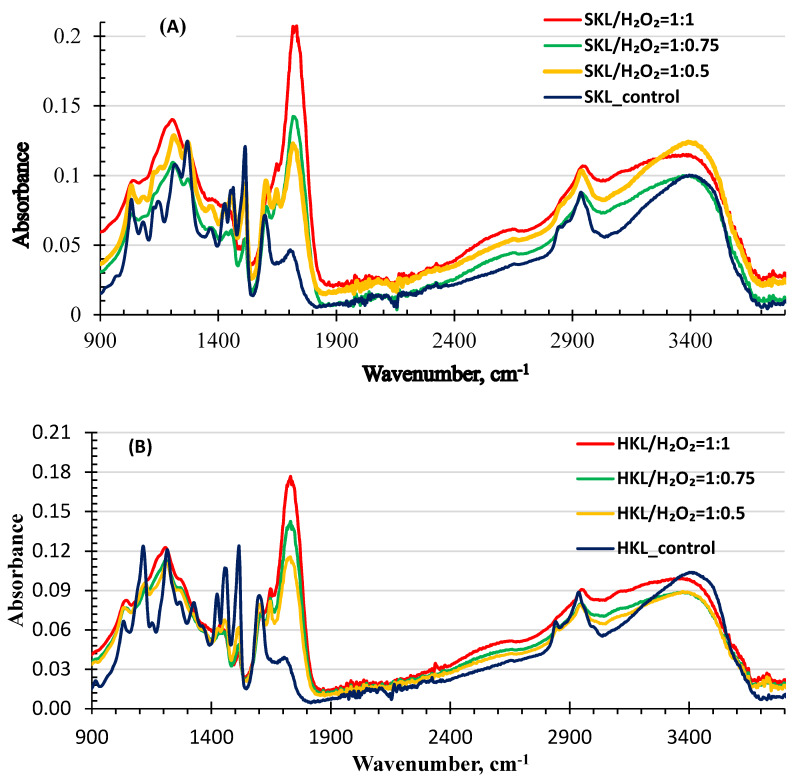
FTIR spectra of the original SKL (**A**), HKL (**B**), and depolymerized lignin products produced at various lignin/H_2_O_2_ mass ratios (1:1, 1:0.75 and 1:0.5).

Figure 3 presents FTIR spectra of the original SKL (Figure 3A) and HKL (Figure 3B), as well as the depolymerized lignin products generated at different lignin/H_2_O_2_ mass ratios (1:1, 1:0.75 and 1:0.5). As shown in these figures, there were five major differences in the spectra of the depolymerized lignin products when compared with those of the original lignins (SKL or HKL), as displayed at wavenumbers 1727, 1600, 1515, 1460 and 1272 cm^−1^. The IR absorbance at wavenumber 1727 (cm^−1^) could be attributed to the non-conjugated C=O stretching vibration for carboxyl groups [36]. The intensity of this IR peak increased rapidly when increasing the amount of H_2_O_2_ in the treatment. This implies that the oxidation of lignin by H_2_O_2_ generated more C=O functional groups such as –COOH in the depolymerized lignin products.

The peaks at 1600 cm^−1^ and 1515 cm^−1^ could be ascribed to C=C in aromatic structures [37]. In the case of HKL, these peaks had reduced intensity in the depolymerized lignin products compared to those in the original lignin, indicating that the oxidative treatment reduced the aromatic structure content of lignin. In the case of SKL, specifically at 1600 cm^−1^, the peak was weak and broad without any significant trends being demonstrated at different lignin/H_2_O_2_ mass ratios. The signal reduction at 1600 cm^−1^ and 1515 cm^−1^ could be seen as suggestive evidence for lignin depolymerization as a result of oxidative ring opening. The band at 1460 cm^−1^ could be ascribed to C–H bending in methylene groups [36]. As seen in this figure, the peaks at 1460 cm^−1^ were of approximately similar intensities for the SKL controls and the de-polymerized lignin samples of SKL at the lignin/H_2_O_2_ mass ratio of 1:0.5. However, this peak was not observed in the de-polymerized SKL samples produced at the lignin/H_2_O_2_ mass ratios of 1:0.75 and 1:1. For HKL, these peaks almost disappeared for the depolymerized lignin samples, indicating that under severe oxidation conditions, the generation of electrophilic hydroxonium ions, HO^+^, could lead to the breaking of double bonds, yielding various aldehyde or ketone groups.

The band at 1270 cm^−1^ was assigned to guaiacyl C–O stretching. The peak intensity decreased in the two depolymerized lignin products (SKL) generated from the oxidative treatments at the lignin/H_2_O_2_ mass ratios of 1:0.75 and 1:1 ratio, suggesting that the oxidative depolymerization of lignin would lead to the cleavage of methoxyl groups, resulting in a reduction in the oxygen content of the de-polymerized lignin products, as suggested previously in Table 3. This peak was not observed for HKL samples.

As shown in Figure 3B, in the HKL sample, syringyl ring breathing was represented by the wavenumber 1330 cm^−1^ [3]. This peak disappeared during the oxidation treatment in all depolymerized lignin samples produced at different lignin to peroxide ratios, suggesting oxidative cleavage of methoxyl groups. Furthermore, the aromatic C–H in-plane deformation in syringyl structures observed at wavenumber 1118 cm^−1^ was observed in the HKL sample, but it was not detected for any of the treated hardwood lignins, again indicating cleavage of methoxyl groups during the oxidative treatment [38].

### 2.4. Molecular Weight Distribution by GPC

**Figure 4:**
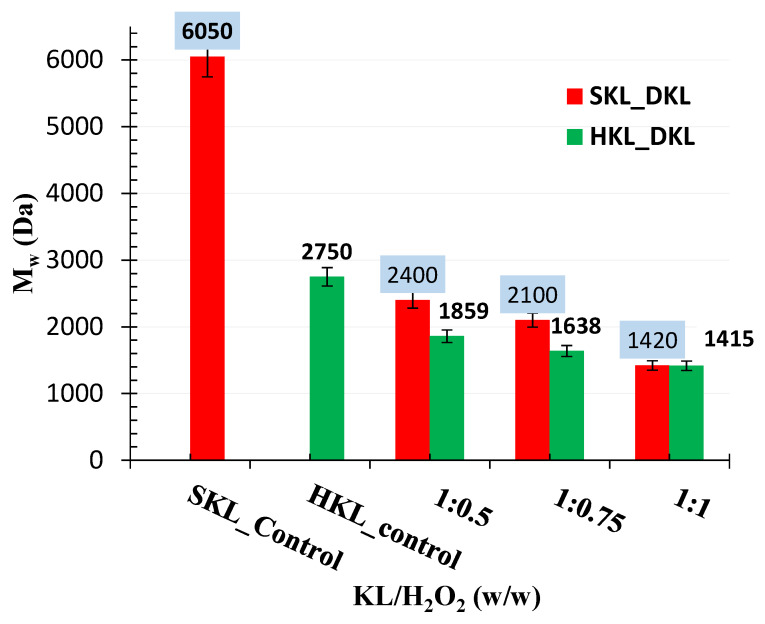
Weight average molecular weight (M_w_) of SKL and HKL, and the de-polymerized kraft lignin (DKL) products at different lignin to peroxide ratios.

The molecular weights and distributions of the original lignins (SKL and HKL) and depolymerized lignin products, after acetobromination, were measured by GPC–UV using a method as described in [39]. Figure 4 displays the weight average molecular weight (M_w_) of SKL and HKL, and the de-polymerized kraft lignin (DKL) products. As clearly shown, the molecular weight of hardwood kraft lignin (M_w_ = 2718 Da) was much lower than that of softwood kraft lignin (M_w_ = 6041 Da), as commonly reported in the literature [3,40]. The error bars indicate the relative standard deviations = 5%.

As also clearly shown in Figure 4, an increase in the H_2_O_2_/KL mass ratio in the oxidative treatment led to a dramatic decrease in the molecular weight, especially for softwood kraft lignin. For instance, for SKL, the M_w_ dropped from 6041 Da to 1400 Da for the DKL from the treatment at a 1:1 H_2_O_2_/KL mass ratio at room temperature for 120 h, while, for HKL, the M_w_ was reduced from 2718 Da to 1415 Da for the DKL from treatment under the same conditions. Despite the slightly higher β-*O*-4 linkage content in hardwood lignin compared to softwood lignin, the rate of cleavage of benzyl ether bonds of syringyl nuclei (rich in hardwood lignin) was found to be slower than that of guaiacyl nuclei (more prevalent in softwood lignin) in acidic media [35].

Thus, in the case of both SKL and HKL, the lignin depolymerization products were of a lower molecular weight compared to the control lignins. Furthermore, their solubility in water at a low pH was remarkably high. As discussed previously, lignin oxidative depolymerization using concentrated H_2_O_2_ could involve the production of phenolic oligomers by cleavage of ether bonds and demethoxylation of syringyl and guaiacyl nuclei. Extensive oxidation could produce ring opening products (e.g., muconic acids and their derivatives) [17,41].

### 2.5. Functional Group Analysis Using ^31^P-NMR

One of the most important functional groups in lignin is the hydroxyl group, especially the free phenolic group, as the physical and chemical properties of lignin and depolymerized lignin are affected by the content of such groups. ^31^P-NMR spectroscopy is a unique tool in the measurement of lignin hydroxyl groups, providing quantitative information for various types of major hydroxyl groups. After sufficient phosphitylation with phosphorous-based reagents, different hydroxyl groups in lignin belonging to aliphatic, carboxylic, guaiacyl, syringyl, p-hydroxyphenyl, catechol as well as guaiacyl groups with carbon substituents at the C5 position, could be readily quantified with ^31^P-NMR spectroscopy [42].

**Figure 5:**
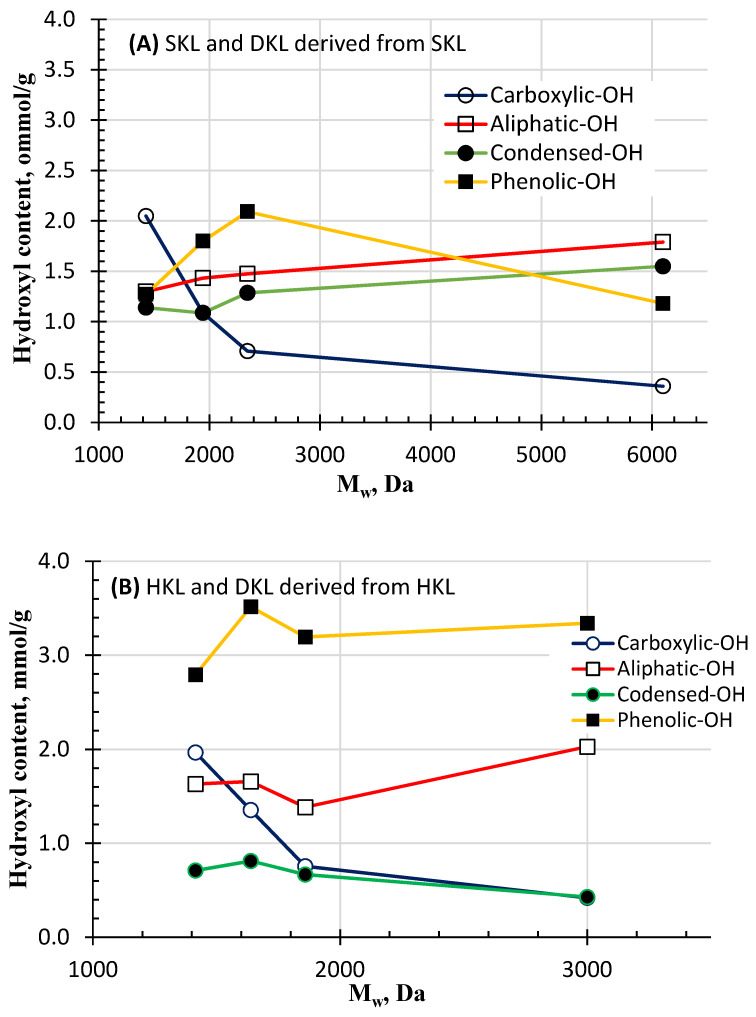
Contents of different hydroxyl functional groups vs. M_w_ for SKL and DKL derived from SKL (**A**) and HKL and DKL derived from HKL (**B**).

Figure 5A,B presents the contents of different hydroxyl functional groups as a function of M_w_ for SKL and HKL. The carboxylic acid group is typically associated with extensive oxidation; hence, among all different types of hydroxyl groups, the carboxylic hydroxyl group content dramatically increased with decreasing M_w_ at a higher H_2_O_2_/lignin ratio. For example, while carboxylic-OH groups existed in both the SKL and HKL controls at relatively low levels (i.e., 0.3–0.4 mmol/g), these levels increased to 2 mmol/g in DKL derived from either SKL or HKL after treatment at the 1:1 H_2_O_2_/lignin mass ratio. This could be attributed to comprehensive oxidation of lignin by H_2_O_2_.

As shown in Figure 5A, the aliphatic hydroxyl group is the dominant hydroxyl group in softwood kraft lignin due to the presence of phenylpropane units in the lignin structure, represented by either the primary-OH (in γ-C atom of the side chain) or the secondary-OH (in α-C atom) groups. Lignin oxidative depolymerization with permanganate and ionic liquid, resulted in a decrease in aliphatic OH and an increase in phenolic hydroxyl groups [43,44].

The reduction in aliphatic OH could be attributed to dehydration of the primary and/or secondary-OH groups [45]. As shown in Figure 5A, the aliphatic OH group content in SKL decreased slightly with increasing oxidation intensity, dropping from 1.5 mmol/g in the original lignin to 1.27 mmol/g in the DKL at an M_w_ of 1420 Da produced at the 1:1 H_2_O_2_/SKL mass ratio. A similar trend was observed with oxidative de-polymerization of hardwood kraft lignin for which the aliphatic OH content dropped from 2 mmol/g for the HKL to 1.6 mmol/g in the DKL at a M_w_ of 1415 Da produced at the 1:1 H_2_O_2_/HKL mass ratio (Figure 5B). The condensed hydroxyl structures in lignin comprise diphenylmethanes, diphenyl ethers and 5,5′-biphenolic moieties [46]. As shown in Figure 5A,B, softwood lignins, which are characterized with a high content of guaiacyl (G) units, have a more condensed and cross-linked structure than hardwood lignins because the C5 position in hardwood lignin is occupied by a methoxyl group. As shown in Figure 5A, for SKL, the total condensed OH groups decreased gradually from 1.55 mmol/g in the original softwood kraft lignin to 1.14 mmol/g in the DKL from the oxidative treatment of SKL at 1:1 H_2_O_2_/SKL mass ratio. This decrease could be explained by the scission of β-*O*-4 linkages that led to non-condensed moieties. On the contrary, the total content of condensed hydroxyl groups in hardwood kraft lignin increased from 0.43 mmol/g in the original HKL to 0.711 mmol/g in the DKL from the oxidative treatment of HKL at a 1:1 H_2_O_2_/SKL mass ratio (Figure 5B). This suggests that during oxidative depolymerization, demethoxylation might have occurred, converting syringyl (S) to guaiacyl (G) structures. Subsequently, repolymerization of the G structure at the C5 position could have taken place, accounting for the increase in the condensed OH structures in DKL. Non-condensed phenolic OH groups in lignin consist of syringyl phenolics, guaiacyl phenolics and *p*-hydroxy phenyl. In softwood lignin, the major non-condensed phenolic hydroxyls are guaiacyl phenolics with a minor amount of *p*-hydroxy phenyl. The total content of phenolic OH groups increased from 1.18 mmol/g in the original SKL to 2.09 mmol/g in the DKL at a 0.5:1 H_2_O_2_/SKL mass ratio, which was likely due to the reduction in condensed OH content (Figure 5A), but it decreased back to 1.27 mmol/g in the DKL at a 1:1 H_2_O_2_/SKL mass ratio. In contrast, the total content of phenolic OH groups in HKL (3.34 mmol/g) was much higher than that of SKL (1.18 mmol/g), as hardwood lignins contain more syringyl phenolics than softwood lignins. The total content of non-condensed phenolic OH groups in HKL dropped to 2.79 mmol/g in the DKL at a 1:1 H_2_O_2_/HKL mass ratio, likely due to oxidative ring cleavage, which was in good agreement with the reduced aromatic structure in the DKLs as shown by the FTIR results (Figure 3).

### 2.6. TGA Analysis

**Figure 6:**
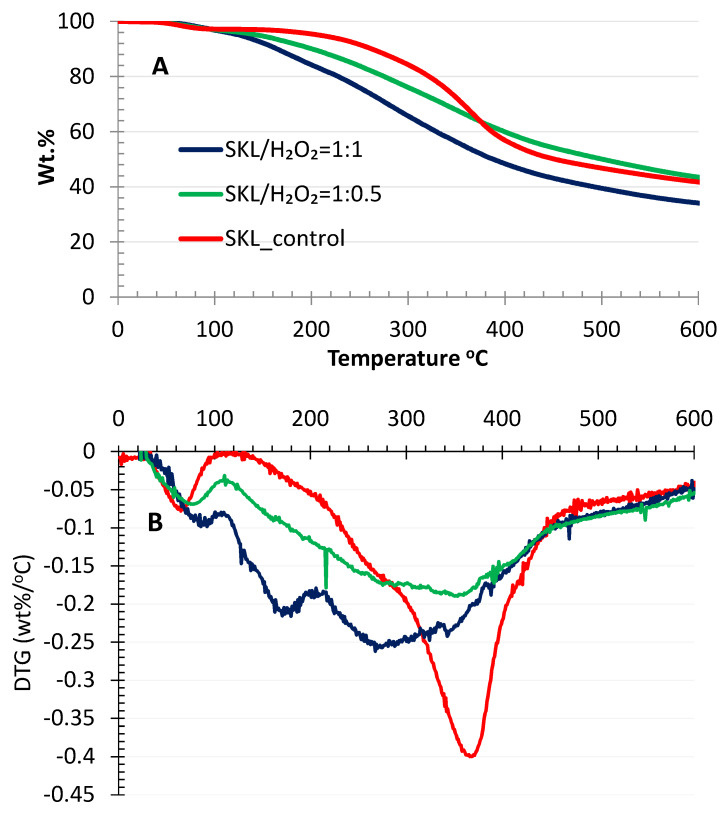
TGA curves (**A**) and DTG plots (**B**) of SKL and its de-polymerized products.

**Figure 7:**
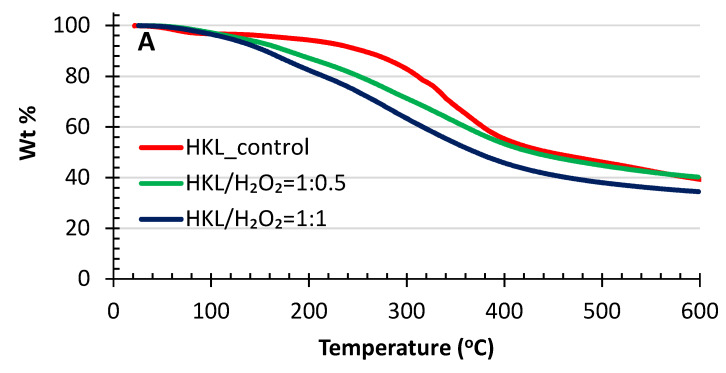

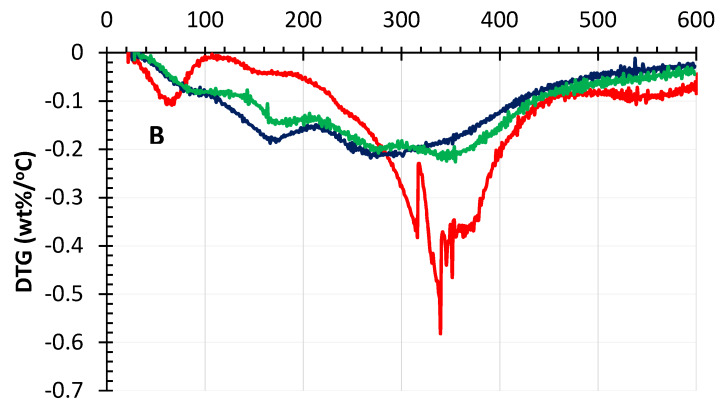
TGA curves (**A**) and DTG plots (**B**) of HKL and its de-polymerized products.

The thermal stability of the original kraft lignins and the depolymerized kraft lignins (DKL) were investigated using thermogravimetric analysis (TGA) plots and the derivative of the TG curves (DTG) under a nitrogen environment, as illustrated in Figure 6 (SKL) and Figure 7 (HKL). The thermal degradation of lignin is a complex process because the structure contains various oxygen functional groups with different decomposition pathways, including competitive and/or consecutive reactions. As shown in Figure 6 and Figure 7, generally both the SKL and HKL lignins started to decompose at 200–300 °C, while the maximum decomposition rate occurred around 350–400 °C. Various aromatic hydrocarbons can form from lignin decomposition, such as phenolics, hydroxyphenolics and guaiacyl-/syringyl-type compounds, most products having phenolic –OH groups [47,48]. The weight loss at temperatures < 100 °C for all samples in both figures could be ascribed to the loss of water contained in the KL or DKL samples [49] as well as volatile sulphur compounds. The lignin decomposition temperatures depend on its molecular structure. Lignins with higher molecular weights incorporating a significant number of intramolecular β-*O*-4 and C–C bonds have a high thermal stability and, as a result, higher decomposition temperatures [50]. Depolymerization of kraft lignins using a low and a high H_2_O_2_/lignin mass ratio produced DKL products with reduced M_w_ (Figure 4). Consequently, the DKL products from either SKL (Figure 6) or HKL (Figure 7) had a much lower decomposition temperature than that of the original lignins. For instance, the initial decomposition temperature and the temperature of maximum decomposition rate of the SKL control with an M_w_ of 6050 Da were 280 and 370 °C, respectively, while for the DKL (M_w_ = 1420 Da) obtained at a H_2_O_2_/lignin mass ratio of 1:1 (Figure 6), these parameters were 173 and 270 °C, respectively. The same conclusions can be drawn when comparing the thermal decomposition temperatures of HKL and DKL (Figure 7). This decrease in decomposition temperatures can be attributed to the greatly reduced M_w_ [51].

### 2.7. Py–GC/MS Analysis

Py–GC/MS analysis was conducted for the DKL products from oxidative de-polymerization of SKL (Table 3) and HKL (Table 4) at room temperature at a 1:1 H_2_O_2_/KL mass ratio. The results of Py–GC/MS analysis could reveal key compounds of the volatile fraction of the DKL samples at 350 °C, identified by their mass spectra by comparison with those appearing in the NIST library. This technique could also provide an idea of the structural components of lignin, which break down at high temperatures to more volatile chemical compounds. It should be noted that the small peaks with an area less than 1.5% of the total area were not included in the Tables. As shown in both Tables, the detectable compounds were mainly aromatic and aliphatic compounds such as guaiacol-type, phenol-type, catechol-type and other oxygen-rich derivatives (carboxylic acids, esters and alcohols). Various types of phenolic compounds can be generated via the cleavage of β-aryl etheric bonds present in both softwood and hardwood samples [52]. Meanwhile, some aliphatic compounds (e.g., 1-butene, 3,3-dimethyl- and adipic acid, di (oct-4-yl ester)) can form from the demethoxylation reaction especially in hardwood samples [53]. The demethoxylation cleavage reaction was confirmed in this work by FTIR analysis (Figure 3).

Moreover, the Py–GC/MS analysis results also confirmed that the SKL and HKL were extensively oxidatively depolymerized by H_2_O_2_ at ambient temperature, producing low-molecular aromatic/phenolic compounds that can be utilized for bio-based phenolic resins, polyurethane materials and dispersants [6,54].

## 3. Discussion

As previously mentioned, the SKL and HKL lignins used in this work were both in the H-form (protonated form) since they were washed with acid, giving a pH value of around 4.0 once suspended in pure water. Thus, in this work, the oxidation reactions were carried out under acidic conditions. Hydrogen peroxide is also a weak acid. Hence, the low pH of the reaction medium is expected to have affected the lignin degradation mechanism in hydrogen peroxide [25]. H_2_O_2_ can act as a nucleophilic or an electrophilic agent depending on the pH of the reaction medium. Under acidic conditions, H_2_O_2_ can form a hydroxonium ion, HO^+^, which is a strongly electrophilic oxidant [55] formed by the reaction of either a mineral acid (e.g., sulfuric acid) or carboxylic acids (e.g., acetic acid, formic acid) with H_2_O_2_ as follows [17,56]:

H_2_O_2_ + H^+^ ⇌ HO^+^ + H_2_O (1)

The electrophilic HO^+^ ion was previously found to be highly reactive towards electron-rich sites such as olefinic, carbonyl and aromatic ring structures (e.g., as found in lignin) [57,58] leading to lignin demethoxylation and β-ether cleavage. As such, depolymerization of lignin can be achieved using an acidic system of hydrogen peroxide via HO^+^ ions. As shown in Equation (1), the formation of HO^+^ ions from H_2_O_2_ is a reversible reaction; therefore, one would expect that high concentrations of the reactant (H_2_O_2_) would favor the formation of HO^+^ ions thereby promoting lignin depolymerization at room temperature [59].

**Scheme 1:**
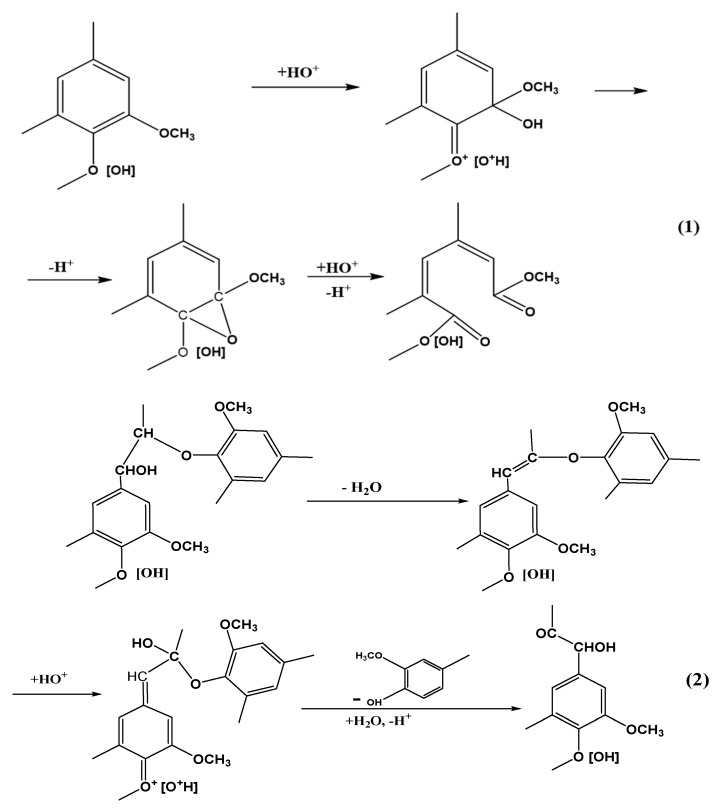
Oxidative ring opening and de-polymerization pathways [55].

Although the exact mechanism of lignin degradation via concentrated H_2_O_2_ has not been reported, the reaction mechanism for the room-temperature H_2_O_2_ depolymerization of acidic kraft lignin may resemble the reaction scheme for the reaction of model compounds with HO^+^ at low pH [55], as illustrated in Scheme 1, involving mainly HO^+^ oxidative ring opening (1) and cleavage of β-aryl ether bonds (2).

## 4. Materials and Methods

### 4.1. Materials

Two types of kraft lignin (KL), softwood kraft lignin (SKL) from a western Canadian mill (West Fraser, Hinton, Alberta, Canada) and a hardwood kraft lignin (HKL) from a central Canadian mill (Resolute, Thunder Bay, ON, Canada) were used in this study. Both kraft lignins were separated from kraft black liquor using the LignoForce^TM^ process previously developed by FPInnovations (Pointe Claire, Quebec, Canada). More specifically, the black liquor was oxidized with molecular oxygen at 80 °C, and then the pH was reduced using CO_2_ and H_2_SO_4_ followed by filtration and washing with dilute H_2_SO_4._ As a result, both lignins produced were in the H-form (protonated lignin), and both substrates, SKL and HKL, when suspended in pure water, had a pH value of around 4.0. The low pH of the reaction medium is expected to affect lignin degradation in hydrogen peroxide [25]. Hydrogen peroxide can act as a nucleophilic or an electrophilic agent depending on the pH of the solution (it is relatively stable under acidic conditions). Thus, in this work, the oxidation reactions were carried out under acidic conditions. Table 5 displays the elemental analysis and the physical properties of SKL and HKL. All other chemicals used in the study, such as 50% hydrogen peroxide, were ACS reagent-grade chemicals purchased from Sigma-Aldrich (Oakville, ON, Canada) and used as received.

### 4.2. Oxidative Treatment of SKL and HKL

The oxidative treatment of kraft lignins, SKL and HKL (dry solid powder), was conducted in a 150-mL beaker under ambient conditions. In a typical run, 6 g (or 4.5 g or 3 g) of 50% H_2_O_2_ was added slowly to 3 g of SKL or HKL on a dry basis, with the ratio of H_2_O_2_/KL being kept at 1:1 *w/w* (or 1:0.75 *w/w* or 1:0.5 *w/w*, respectively). The mixture was then uniformly mixed (manual mixing with a spatula) at ambient temperature to obtain a homogenous slurry. The mixture was then kept inside a fume hood at ambient temperature without a cover, or in an oven under controlled temperature (not exceeding 35 °C) if a shorter reaction time was desired. The mass was monitored over time until a final dry product was obtained. The recovered modified KL was air dried for 24 h to determine the yield. The yield was determined based on the percentage of the dry mass of the modified KL to the dry mass of the initial KL.

### 4.3. Product Characterization

#### 4.3.1. Elemental analysis

The elemental composition of the samples, i.e., CHNS (carbon, hydrogen, nitrogen and sulfur), was determined using a CHNS-O flash elemental analyzer 1112 series (Thermo, Waltham, MA, USA), using 2,5-bis (5-tert-butyl-benzoxazol-2-yl) thiophene (BBOT) as the calibration standard. The oxygen content (%) was calculated by difference (=100% − C% − H% − N% − S% − Ash%).

#### 4.3.2. GPC Analysis

The average molecular weights and molecular weight distributions of SKL and HKL, and the depolymerized lignin products were measured using a Waters GPC–UV (Waters, Mississauga, ON, Canada) (gel permeation chromatographic system with an on-line UV detector at 270 nm) equipped with a Waters Styragel HR1 column at 40 °C, using THF as the eluent at 1 mL min^−1^ and linear polystyrene standards for molecular weight calibration. All the samples were derivatized by acetobromination before injecting into GPC.

#### 4.3.3. FTIR Analysis

Fourier-transform infrared spectroscopy (FTIR) was employed to investigate the functional group structures of the dry samples of SKL (control) and HKL (control) and the depolymerized lignin products and their changes during the de-polymerization process. The FTIR analysis was performed on a Nicolet-6700 FTIR with a universal ATR accessory (Perkin-Elmer, Waltham, MA, USA).

#### 4.3.4. ^31^P-NMR

The hydroxyl group content of the lignin and modified lignin samples was measured using quantitative ^31^P-NMR spectroscopy (Varian Inova, Palo Alto, CA, USA). The samples were derivatized with 100 μL of 2-chloro-4,4,5,5-tetramethyl-1,3,2-dioxaphospholane (TMDP). Derivatized samples (30–40 mg) were dissolved in 500 μL of anhydrous pyridine and deuterated chloroform (1.6:1, *v*/*v*) and mixed with 100 L of a solution of *N*-hydroxy-5-norbornene-2,3-dicarboxylic acid imide (10 mg mL^−1^) and chromium (III) acetylacetonate (5 mg mL^−1^) as internal standard and relaxation agent. The solution was thoroughly mixed and transferred to a sealed 5 mm NMR tube. All NMR experiments were carried out at 298 K on a Varian Inova 500 NMR Spectrometer [60]. ^31^P-NMR spectra were acquired using an inverse-gated decoupling pulse sequence with a 90-pulse angle, 25 s relaxation delay, and 256 scans.

#### 4.3.5. TGA Analysis

Thermogravimetric analysis (TGA) of the lignin and depolymerized lignin products was performed using a PerkinElmer Pyris 1,1 TGA in a nitrogen atmosphere (PerkinElmer, Waltham, MA, USA). The dry samples weighing approximately 10 mg were heated in an N_2_ flow at 20 mL/min from 25 °C to 600 °C at 10 °C/min. The weight loss, percent and the rate of weight loss (DTG) of the samples were recorded.

#### 4.3.6. Py–GC/MS

Pyrolysis–gas chromatography–mass spectroscopic analysis was conducted on an Agilent 6890B GC coupled with a 5973A MSD using 30 m × 0.25 mm × 0.25 µm DB-5 columns with temperature programming as follows: 1 min hold at an initial temperature of 50 °C followed by a 30 °C min^−1^ ramp to the final temperature of 350 °C with a 1 min hold. Py–GC/MS analysis was done using the double-shot mode. Pyrolysis of samples (∼100 μg) was performed with a py2020i microfurnace pyrolyzer/AS1020 autosampler (Frontier Laboratories, Koriyama, Fukushima, Japan). The compounds were identified by comparing their mass spectra with those in the NIST library.

## 5. Conclusions

This work successfully demonstrated a cost-effective approach for converting kraft lignin into low molecular weight compounds at close to 100% yield by oxidative degradation of lignin slurries with concentrated H_2_O_2_ at ambient temperature under acidic conditions. The obtained depolymerized kraft lignin (DKL) products were comprehensively characterized by GPC–UV, FTIR, ^31^P-NMR, TGA, Py–GC/MS and elemental analysis. The characterization results suggest effective depolymerization of softwood and hardwood lignins. The DKL products have a M_w_ of around 1400 Da with a high phenolic and carboxylic group content when produced at a 1:1 H_2_O_2_/KL mass ratio. The Py–GC/MS results disclosed the presence of highly oxygenated fragments with functional groups such as phenols, carboxylic acids, esters, ketones and aldehydes in the DKL products. The novelty of this oxidative lignin de-polymerization approach is that it can be conducted at room temperature in the absence of a catalyst when acid-washed kraft lignins are used as the substrate. Furthermore, the proposed process can readily be scaled-up to the industrial level, and the DKL produced can be used directly in applications without any further purification.

## Figures and Tables

**Table 1:** General compositions of softwoods and hardwoods and their lignin linkages [27,28].

	Softwood	Hardwood
**Chemical Composition (wt%)**		
Cellulose	40–44	40–44
Hemicellulose	30–32	15–35
Lignin	25–32	18–25
Extractives	5	2
**Lignin Linkage Types (number/100 phenylpropane units)**		
β-*O*-4	43–50	50–65
α-*O*-4	6–8	4–8
β-5 + α-*O*-4	9–12	4–6
β–β	2–4	3–7
5–5′	10–25	4–10
4-*O*-5′	4	6–7
β-1	3–7	5–7
C-6, C-2	3	2–3

**Table 2:** Elemental compositions of the original SKL, HKL, and depolymerized lignin samples at various lignin/H_2_O_2_ mass ratios (1:1, 1:0.75 and 1:0.5).

Elemental Composition, wt% (d.b.)	Original Lignin	De-Polymerized Lignins			Original Lignin	De-Polymerized Lignins		
	SKL	1:1	1:0.75	1:0.5	HKL	1:1	1:0.75	1:0.5
C	65.2	50.5	53.9	57.4	63.2	47.7	49.9	52.5
H	5.5	4.9	4.7	5.0	5.41	3.74	3.87	4.3
N	0.2	0	0.06	0.06	0.02	0.08	0.06	0.08
S	1.51	1.79	1.27	1.29	2.25	1.9	1.9	1.9
Ash ^1^	0.83	0.83	0.83	0.83	0.44	0.44	0.44	0.44
O ^2^	26.8	42.0	39.2	35.4	28.5	46.1	43.8	40.8

^1^ Assuming the depolymerized lignin has the same ash content as the original lignin; ^2^ by difference.

**Table 3:** Py–GC/MS analysis results for the DKL product from oxidative de-polymerization of SKL at room temperature at a 1:1 H_2_O_2_/SKL mass ratio.

Pk#	RT	Area%	Library/ID	M_w_	Qual
1	0.36	2.35	Acetic acid, methyl ester	74	72
2	0.84	7.5	Acetic acid	60	86
3	3.71	1.6	Furfural	96	91
4	5.44	1.7	Propanedioic acid, dimethyl ester	132	83
5	8.37	10.5	Phenol, 2-methoxy-(guaiacol)	124	97
6	10.63	3.01	2-Methoxy-5-methylphenol	138	90
7	10.79	4.68	1,2-Benzenediol-(catechol)	110	95
8	15.93	3.67	1,4-Benzenediol, 2-methoxy-	140	95
9	17.6	2.53	Benzoic acid, 4-hydroxy-3-methoxy-methyl ester	182	96
10	17.78	2.07	Homovanillyl alcohol	168	80
11	18.32	10.1	3-Hydroxy-4-methoxybenzoic acid	168	95
12	21.75	4	9,10-Anthracenedione	208	95
13	35	2.21	Urs-12-ene	410	83

**Table 4:** Py–GC/MS analysis results for the DKL product from oxidative de-polymerization of HKL at room temperature and 1:1 H_2_O_2_/HKL mass ratio.

Pk#	RT	Area%	Library/ID	M_w_	Qual
1	0.23	11.55	Acetic acid, methyl ester	74	64
2	0.61	14.13	Acetic acid	60	90
3	3.7	6.91	Furfural	96	94
4	5.45	8.93	Propanedioic acid, dimethyl ester	132	83
5	9.45	3.64	1-Butene, 3,3-dimethyl-	84	50
6	15.92	2.69	1,4-Benzenediol, 2-methoxy-	140	80
7	16.44	4.27	2-Naphthyl methyl ketone	170	22
8	17.6	5.48	Benzoic acid, 4-hydroxy-3-methoxy-methyl ester	182	94
9	17.77	2.07	2-Propanone, 1-(4-hydroxy-3-methoxyphenyl)-	180	80
10	18.18	4.5	Benzoic acid, 4-hydroxy-3-methoxy-	213	93

**Table 5:** Elemental analysis and the physical properties of softwood kraft lignin (SKL) and hardwood kraft lignin (HKL).

	SKL	HKL
Elemental analysis (wt% d.b.) ^1^		
C	65.2	63.2
H	5.52	5.41
N	0.20	0.02
O ^2^	26.7	28.7
Ash ^3^	0.83	0.44
Total S	1.51	2.25
M_w_, UV detector (Da)	6041	2718
PDI ^4^, UV detector	3.3	3.0

^1^ On dry basis; ^2^ by difference; ^3^ ashing in air at 575 °C; ^4^ polydispersity index (PDI = M_w_/M_n_).
